# Study on the multi-stage instability mechanism of the Wachangwan landslide in Gaoxian County, Sichuan, China

**DOI:** 10.1038/s41598-025-93306-9

**Published:** 2025-03-12

**Authors:** Hao Yang, Na Wang, Xingyu Wen, Min Huang, Jian Wan, Xue Huang

**Affiliations:** 1 State Key Laboratory of Geohazard Prevention and Geoenvironment Protection, Chengdu University of Technology, Chengdu, 610059 Sichuan China; 2 Sichuan College of Architectural Technology, Deyang, 618000 Sichuan China; 3 The 12th Geological Brigade of Sichuan, Yibin, 644002 Sichuan China

**Keywords:** Landslide, Multistage Sliding, Evolution Characteristics, Numerical Simulation, Stability Analysis, Natural hazards, Civil engineering

## Abstract

Landslides are one of the most common natural disasters worldwide. On September 27, 2020, a large-scale landslide occurred in the Tianzhulin area of Huangni Village, Wenjiang Town, Gao County, known as the Wachangwan Landslide. Through field investigations and UAV aerial photography, the causes and deformation processes of the Wachangwan landslide were thoroughly revealed. Additionally, the displacement and deformation of the landslide were analyzed using the discrete element method. The key findings are as follows: The Wachangwan landslide is a typical multistage landslide. Prolonged heavy rainfall induced sliding in the front part of the landslide body, which dragged the rear, less stable soil mass, further exacerbating deformation and eventually forming a multistage landslide. The non-sliding zone is located between zones III-1 and III-2, influenced by pressure from adjacent sliding zones. However, based on rainfall data and displacement monitoring, the non-sliding zone remains generally stable at present. Using the discrete element method, the deformation mechanism and subsequent evolution of the Wachangwan landslide under rainfall conditions were analyzed. The landslide exhibited a failure mode characterized by traction, tensile fracturing, and sliding. A stability calculation method for multistage landslides under rainfall conditions was established, combining the traditional transfer coefficient method and the multistage landslide effects. By analyzing the current stability of the Wachangwan landslide, the results provide a novel approach for evaluating the stability of multistage landslides.

## Introduction

Landslides, as a typical geological disaster, are challenging to predict both spatially and temporally^1,2^. In Southwest China, due to unique geological structures and climatic conditions, landslide disasters occur frequently^3^. Slope instability is typically controlled by a combination of geological, hydrological, climatic, human engineering activities, and vegetation cover conditions^4^, resulting in diversity and complexity in landslide development mechanisms and failure modes^5^. Therefore, studying the development mechanisms and failure modes of landslides not only provides a scientific basis for landslide prevention and early warning but also minimizes the impact of landslides on human activities.

Multistage landslides refer to landslides in which the same sliding mode repeatedly occurs within the same landslide body, forming multiple sliding surfaces^5^. The common failure modes of this type of landslide mainly include two types: (1) the stepwise traction-type multi-stage instability, characterized by unloading at the front edge triggering rearward sliding, forming a stepwise traction effect; (2) the thrust-type multi-stage instability, where the rock and soil mass in the middle and rear of the slope first slide, subsequently pushing the middle and front parts, leading to deformation. While the sliding modes of individual landslides have been extensively studied, the repetitive nature of multistage landslides adds complexity to the failure mechanisms, making the deformation mechanisms of such landslides a critical focus of research. Extreme and prolonged rainfall is a major triggering factor for landslides globally. Depending on the mechanisms by which water contributes to slope instability, landslides can be categorized into four types: buoyancy weight loss landslides, hydrodynamic pressure landslides, rainfall-induced landslides, and mixed-type landslides^6,7^. Kirschbaum et al.^8^ used the Global Landslide Catalog (GLC) and 13 years of TRMM satellite rainfall data to quantitatively evaluate the correlation between global rainfall and landslide occurrences. The results indicated a positive correlation between satellite rainfall data and the number of landslides and fatalities in landslide-prone regions. Nguyen et al.^9^ investigated the extreme rainfall event on February 20, 2010, on Madeira Island in the North Atlantic, finding that it triggered numerous shallow landslides and a series of cascading geological disasters, causing significant damage to coastal residential areas. Vase et al.^10^ developed a new probabilistic temporal model and Dynamic Hazard Index based on an improved extreme rainfall-induced landslide index. This model was used to analyze the disaster evolution process of landslides triggered by extreme rainfall in Gangwon Province in July 2006. Kirschbaum et al.^11^ utilized a new landslide disaster dataset and satellite-derived global precipitation data to investigate the potential impact of future changes in extreme precipitation on landslide activity in Asia’s mountainous regions.

In practical engineering, complex geological conditions and diverse working scenarios complicate the triggering mechanisms of landslides, making it challenging to evaluate landslide stability quickly and accurately. Researchers typically employ numerical simulation methods and analytical approaches to analyze landslide stability. Methods for analyzing the stability of multistage landslides can be broadly categorized into four types: Conventional Newmark Method: Used to analyze the stability of slopes with multiple potential sliding surfaces^12^. Numerical Simulation: This involves building geological models, performing local strength reduction to search for multistage sliding surfaces, and calculating the stability factor using limit equilibrium methods^13–16^. Reliability Analysis: Utilizes modern nonlinear science, information science, Monte Carlo simulations, and other probabilistic statistical methods to analyze the reliability of multistage landslides^17,18^. Limit Equilibrium Analysis: Based on static equilibrium principles, this method examines the forces acting on landslides in different failure modes to determine stability through the relationship between resisting and driving forces^19,20^. The transfer coefficient method, as an important technique in limit equilibrium analysis, has been widely recognized and applied in practical engineering fields due to its simplicity in calculation and intuitive principles. Moreover, many scholars have made in-depth improvements to the transfer coefficient method to better align it with actual engineering conditions, thereby enhancing its accuracy and practicality. For example, Zhang et al.^21^ modified the transfer coefficient method by fully considering the impact of dynamic water pressure, which improved its applicability and accuracy when calculating the stability coefficient of riverbank slopes. Zhang et al.^22^ applied methods such as uniform design, transfer coefficient method, genetic algorithms (GA), and artificial neural networks (ANN) for a comprehensive and in-depth optimization analysis of slope cutting schemes for real landslides. Furthermore, Yang et al.^23^ improved the stability calculation model for landslides in loose accumulation bodies under the coupling effects of earthquakes and rainfall, based on the unbalanced thrust method and genetic algorithm (GA) model, while considering complex factors like seismic inertial force and seepage force. This improvement not only enhanced the calculation accuracy and reliability of the model but also provided strong technical support for the prediction and prevention of landslide disasters.

In summary, researchers have conducted extensive studies on the stability and failure mechanisms of multistage landslides from various perspectives. However, insufficient consideration has been given to the impact of rainfall conditions and hydrostatic pressure under different failure states, resulting in significant discrepancies between calculation results and actual conditions. To address this issue, the study focuses on the large-scale multistage landslide that occurred in the Tianzaolin area of Huangni Village, Wenjiang Town, Gao County, on September 27, 2020. Based on a detailed geological field investigation, the deformation evolution mechanism of the landslide was revealed through a zonal analysis of deformation degrees and an examination of deformation signs and developmental features. Further, the discrete element numerical simulation method was applied to conduct an in-depth analysis of the deformation process of the landslide. Finally, a stability calculation model for multistage landslides under rainfall conditions was developed using a transfer coefficient method, and the stability of the Wachangwan landslide under rainfall conditions was evaluated.

## Study area

### Landslide overview

**Fig. 1:**
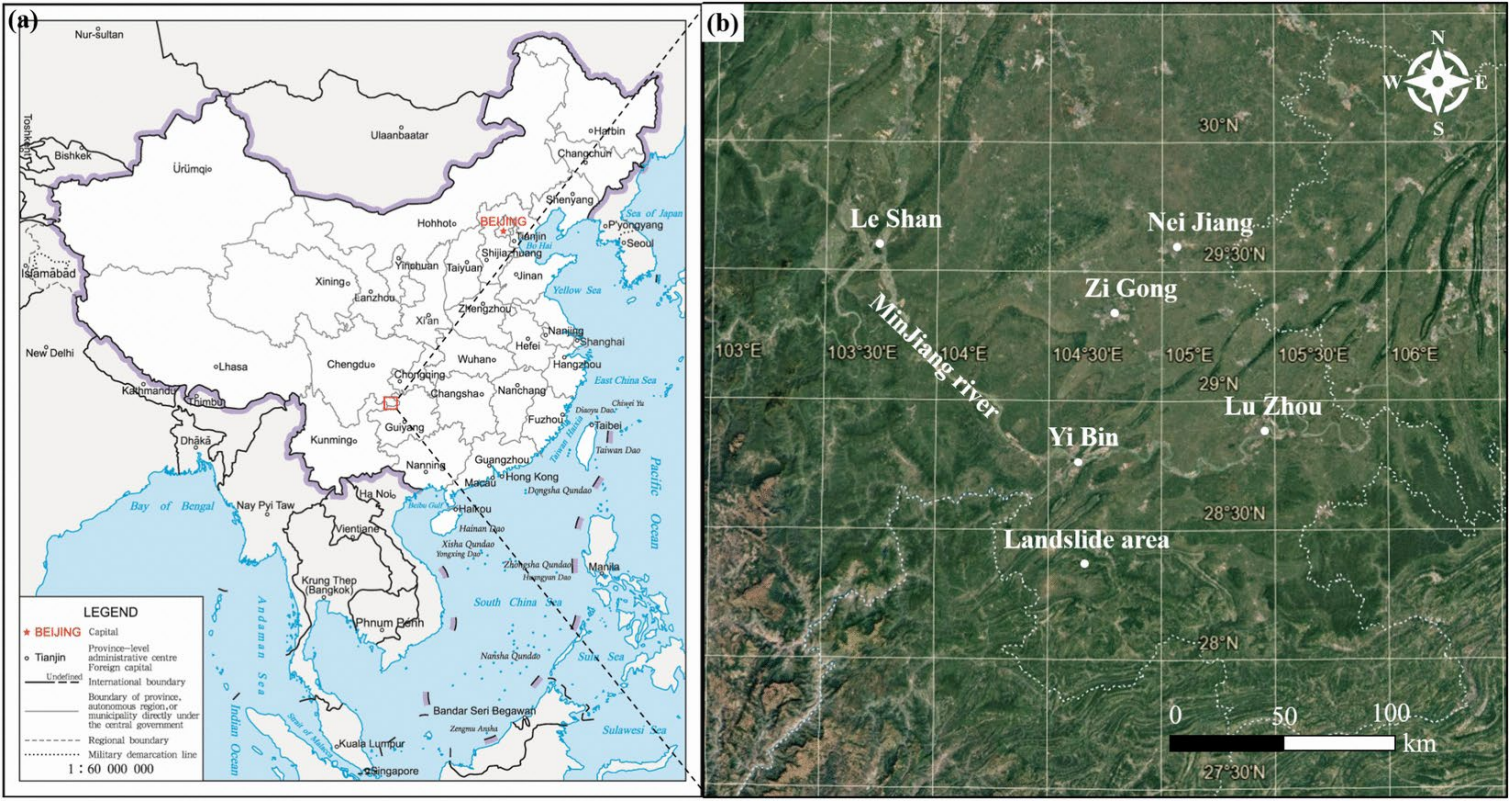
Geographical location of Wachangwan landslide. a Location within China (Photo from Standard Map Service: http://bzdt.ch.mnr.gov.cn/**);** b Location within Yibin City. The map was prepared by Hao Yang in Google Earth (https://earth.google.com/).

**Fig. 2:**
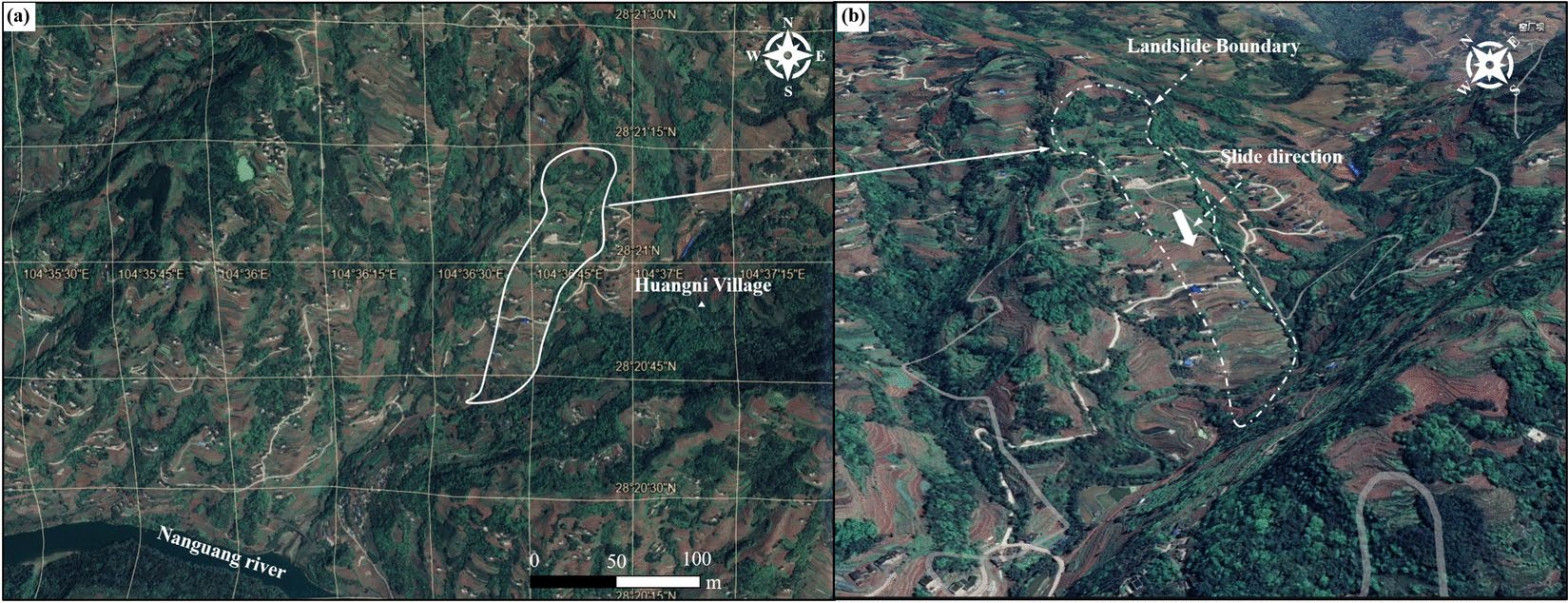
Landslide Boundary and Surrounding Topography and Geomorphology Map. **a** Landslide boundary; **b** Landforms around the landslide. These two maps were prepared by Hao Yang in Google Earth (https://earth.google.com/).

The Wachangwan landslide is located in Huangni Village, Wenjiang Town, Gao County, Yibin City, Sichuan Province, China (Figs. 1 and 2). Before the Wachangwan landslide event occurred, the slope was primarily composed of a large, unstable accumulation of colluvial and residual deposits. Based on field investigations and meteorological data, it was observed that the rainfall in Gaoxian County significantly increased from August to September 2020 compared to previous years. Particularly, during the period from September 17 to September 27, the landslide area experienced continuous heavy rainfall, which led to prolonged surface water infiltration. This continuous infiltration caused progressive deformation of the slope soil and a gradual weakening of its internal mechanical properties. Ultimately, on the afternoon of September 27, under the combined effects of self-weight and cumulative prolonged rainfall, the slope experienced a sliding deformation.

**Fig. 3:**
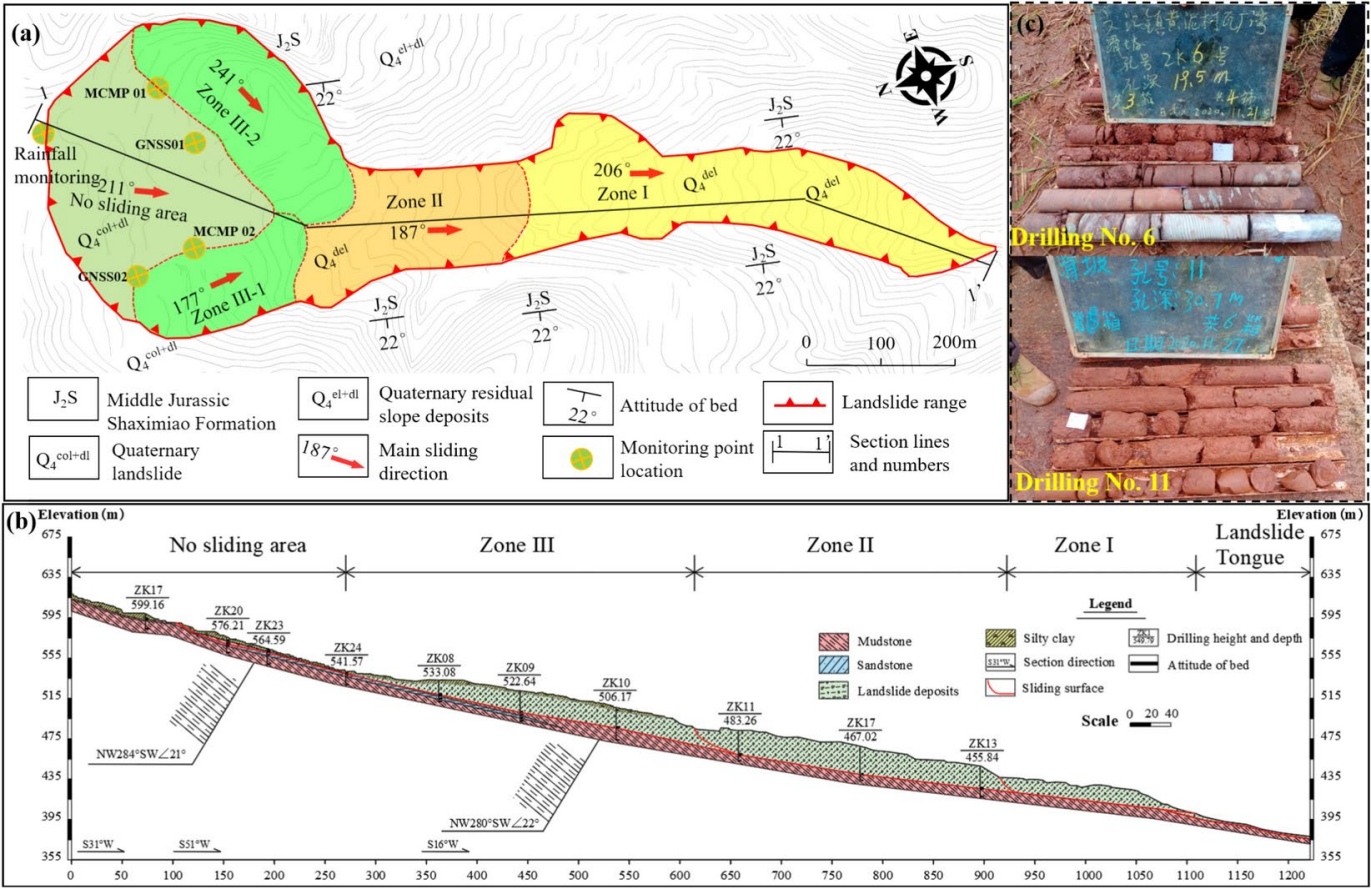
**a** Plan View of the Wachangwan Landslide; **b** Main Cross-Section of the Wachangwan Landslide; **c** Material Composition Revealed by Boreholes No. 6 and No. 11.

The Wachangwan landslide is approximately 1,300 m long, 130–300 m wide, with a total area of about 0.25 square kilometers, and an affected area of approximately 0.3 square kilometers (Figs. 3a and 3b).

The landslide has a general north-to-south slope, with a primary sliding direction of 187°. The sliding body has a thickness of 5–28 m, a volume of approximately 1.743 × 10⁶ cubic meters, and is classified as a large soil landslide. The landslide has a long, narrow strip-shaped plan view. The lateral boundaries are defined by ridges, the rear boundary is marked by through-going tensile cracks in the mid-to-upper slope, and the front boundary is delineated by steep free faces.

The exposed strata in the landslide area belong to the Middle Jurassic Shaximiao Formation, characterized by interbedded thin-layered sandstone and thick-layered mudstone. The bedding orientation is 190°∠22°. The steep slope morphology, influenced by lithology and geological structures, often forms step-like features.

The topography of the landslide area is predominantly high in the north and low in the south, with the highest elevation at 680 m and the lowest at 410 m, resulting in a relative height difference of 270 m. The front portion of the slope is steeper, while the middle and rear sections are relatively gentler, with an overall slope angle of approximately 20° and local gradients reaching up to 30°. The slope orientation aligns with the main sliding direction at 187°.

### Composition and structure of the landslide body

Based on field investigations and drilling measurements, the Wachangwan landslide body is composed of Quaternary Holocene colluvium with a thickness ranging from 5.0 to 28.0 m. The structure is loose, primarily consisting of gravel and silty clay, with gravel content ranging from 50 to 60%. The material also includes a small proportion of boulders and isolated large stones, predominantly sandstone, with particle sizes generally between 2 and 15 cm (Fig. 3c).

The sliding zone soil is a 2 to 4-m-thick, heavily weathered weak layer, appearing purplish-red and composed mainly of breccia and silty clay. The sliding bed is formed by the Middle Jurassic Shaximiao Formation (J₂s), characterized by reddish-brown, medium-thick bedded mudstone. The strata orientation is 190°∠22°, and the surface rock mass is severely weathered, with well-developed fractures, leading to highly fragmented rock.

The landslide body is rich in groundwater, with a shallow water table that deepens gradually from the downstream to the upstream sections. The groundwater system primarily receives recharge from atmospheric precipitation and distant sources, with runoff flowing downstream. Fracture water in the bedrock is mainly hosted in the sandstone of the Middle Jurassic Shaximiao Formation. It is influenced by precipitation and remote groundwater recharge, flowing along joint and fracture networks, with limited discharge in low-lying areas and rare spring exposures.

Field investigations revealed the presence of paddy fields in the central and rear parts of the original landslide body, along with two gullies. These gullies typically lack surface water flow but collect surface runoff from the rear edge of the landslide after heavy rainfall. This water flows along the slope or accumulates in the gullies before discharging at the slope toe, providing favorable conditions for landslide sliding and deformation.

### Deformation characteristics of the landslide zoning

Based on the severity of deformation, the landslide area is divided into sliding zones and non-sliding zones. The sliding zone is further subdivided into four regions—Zone I, Zone II, Zone III-1, and Zone III-2—based on geological characteristics, shear-out locations, deformation features, and deformation chronology observed during field investigations.

#### Description of Zone I

**Fig. 4:**
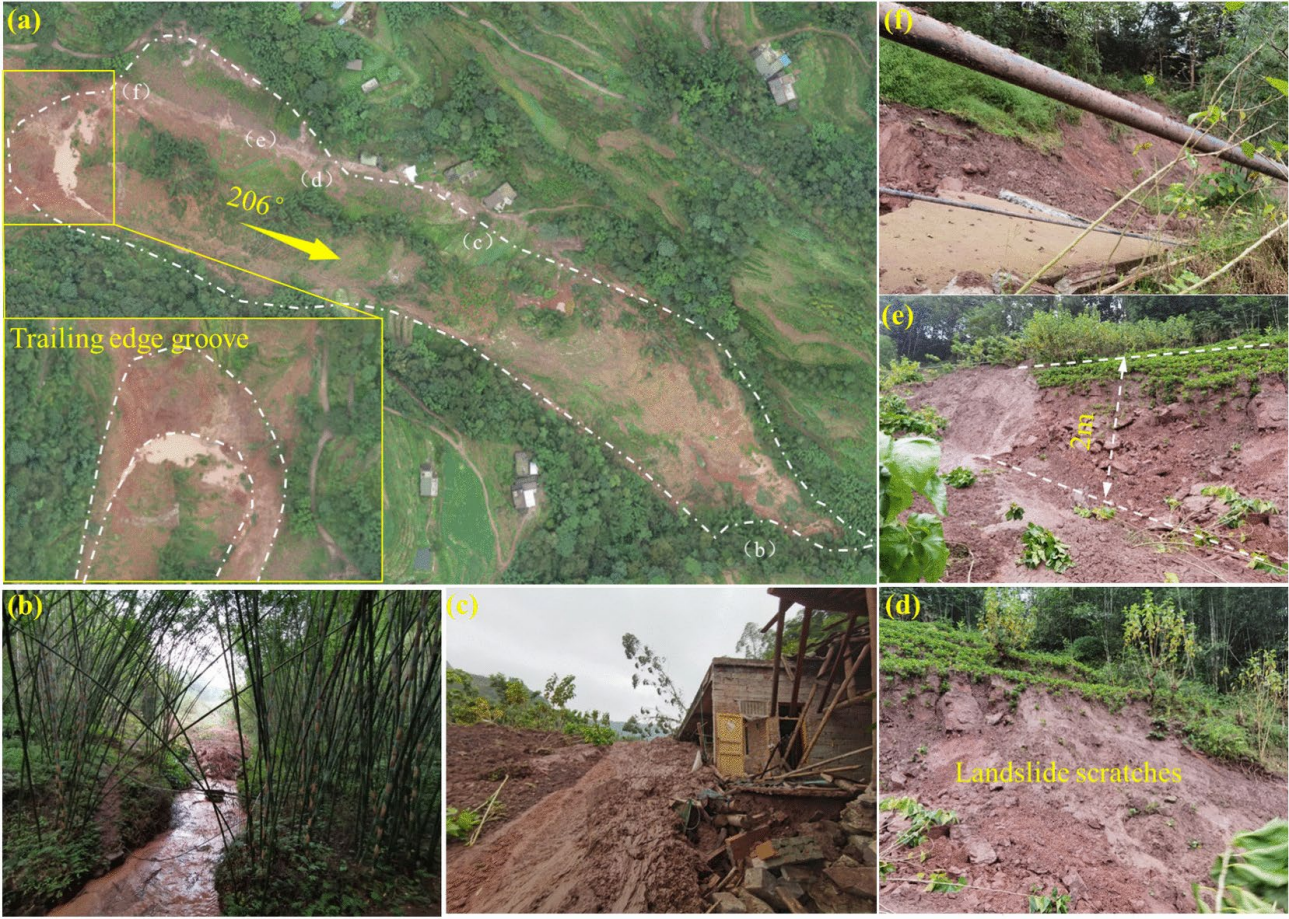
**a** Overview of Zone I; **b** Landslide Tongue; **c** Damage to Houses on the Side of the Landslide; **d** Scratches on One Side of the Landslide; **e** Steep Scarp Formed After Sliding; **f** Road Damage at the Rear Edge of Zone I.

The overall view of Zone I is shown in Fig. 4a. Its plan view appears "tongue-shaped," with a main sliding direction of 206°. The terrain slope is between 10° and 13°, with steep slopes exceeding 30° at the front edge. The boundaries of Zone I are defined by steep cliffs at the front, ridge lines on both sides, a rear edge elevation of approximately 510 m, and a front edge elevation of about 420 m, resulting in a relative height difference of 90 m.

Zone I spans approximately 450 m in length and 100 m in average width. The sliding body exhibits a thin-front and thick-rear profile, with a thickness of 13 to 28 m (average ~ 20 m) and a volume of around 9 × 10^5^ m^3^. The landslide is large-scale, with significant sliding material moving along the channel direction and accumulating in flatter regions, forming a landslide tongue approximately 120 m in length (Fig. 4b). The right boundary of Zone I shows clear scuff marks (Fig. 4d), while severe house damage is observed along the left boundary (Fig. 4c). Subsidence at the left side of the rear edge formed a steep cliff approximately 2 m high (Fig. 4e), causing road breakage at the rear edge (Fig. 4f). Additionally, the rear edge of the landslide has developed a tensile subsidence trench approximately 100 m wide, 50 m long, and 15 m deep, forming the front edge of Zone II.

#### Description of Zone II

**Fig. 5:**
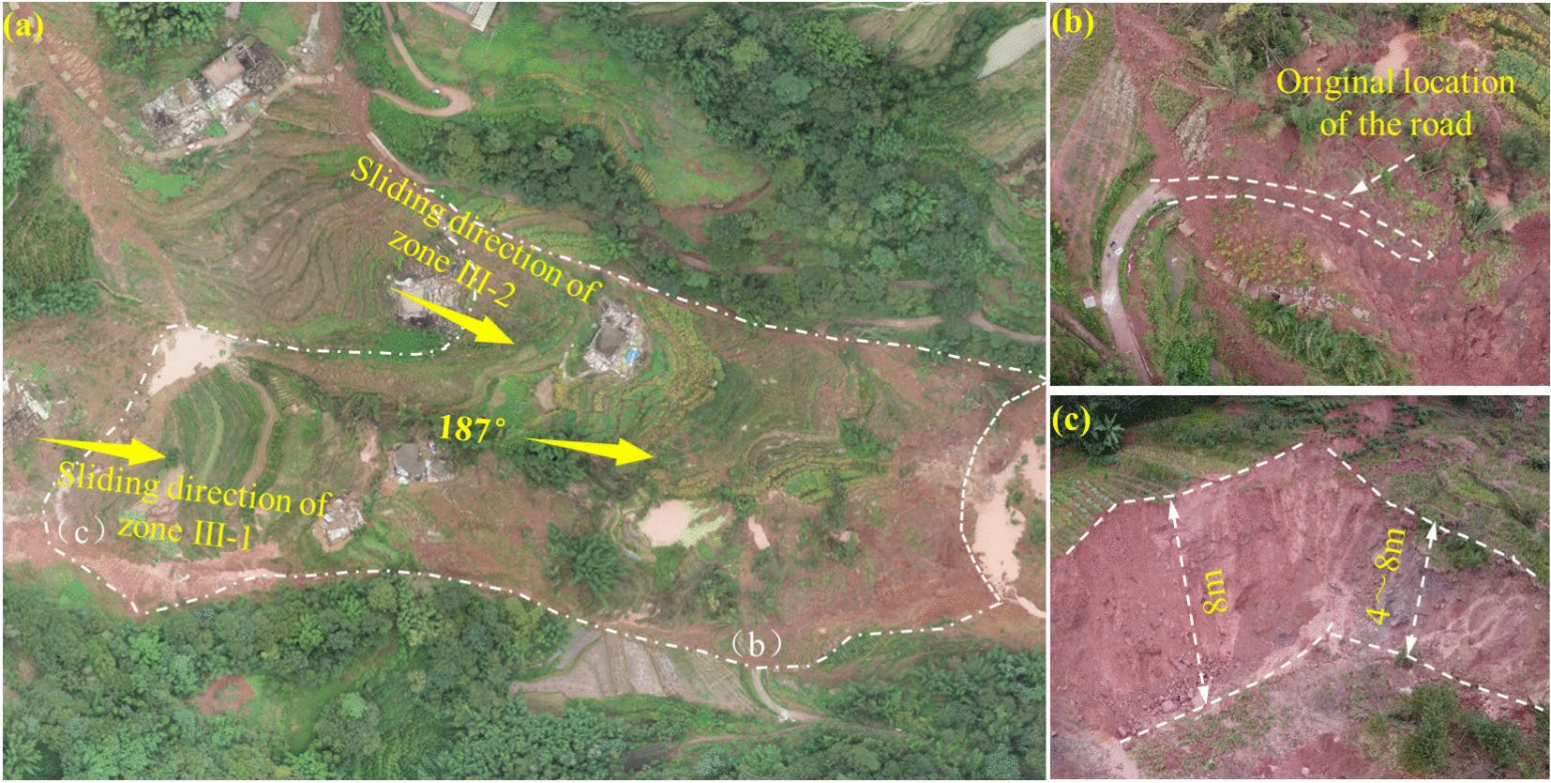
**a** Overview of Zone II; **b** Road Damage at the Boundary of Zone II; **c** Steep Scarp at the Front Edge of Zone III-1.

Figure 5a provides a comprehensive view of Zone II, which exhibits a "strip-like" planar shape. The zone spans approximately 300 m in length and has an average width of 120 m. The sliding body features a thickness profile that is thicker in the middle and thinner at the front and rear, with a thickness range of 15–20 m (average ~ 17 m) and a volume of 2.12 × 10^5^ m^3^. The main sliding direction is 187°, and the terrain slope ranges from 10° to 12°.The front boundary of Zone II is defined by the steep back wall formed after the sliding in Zone I, while the rear boundary corresponds to the transition zone between steep and gentle slopes at the front edge of Zone III. Ridge lines serve as the lateral boundaries. The elevation at the rear edge is approximately 550 m, while the front edge elevation is about 410 m, resulting in a relative height difference of 140 m.

 During the sliding process, Zone II caused damage to the road along its right boundary (Fig. 5b). Additionally, at the rear of Zone II, located at the front edge of Zone III-2, a tensile subsidence trench developed. This trench is approximately 90 m wide, 40 m long, and 4–8 m deep (Fig. 5c).

#### Description of Zone III and the Non-sliding Zone

**Fig. 6:**
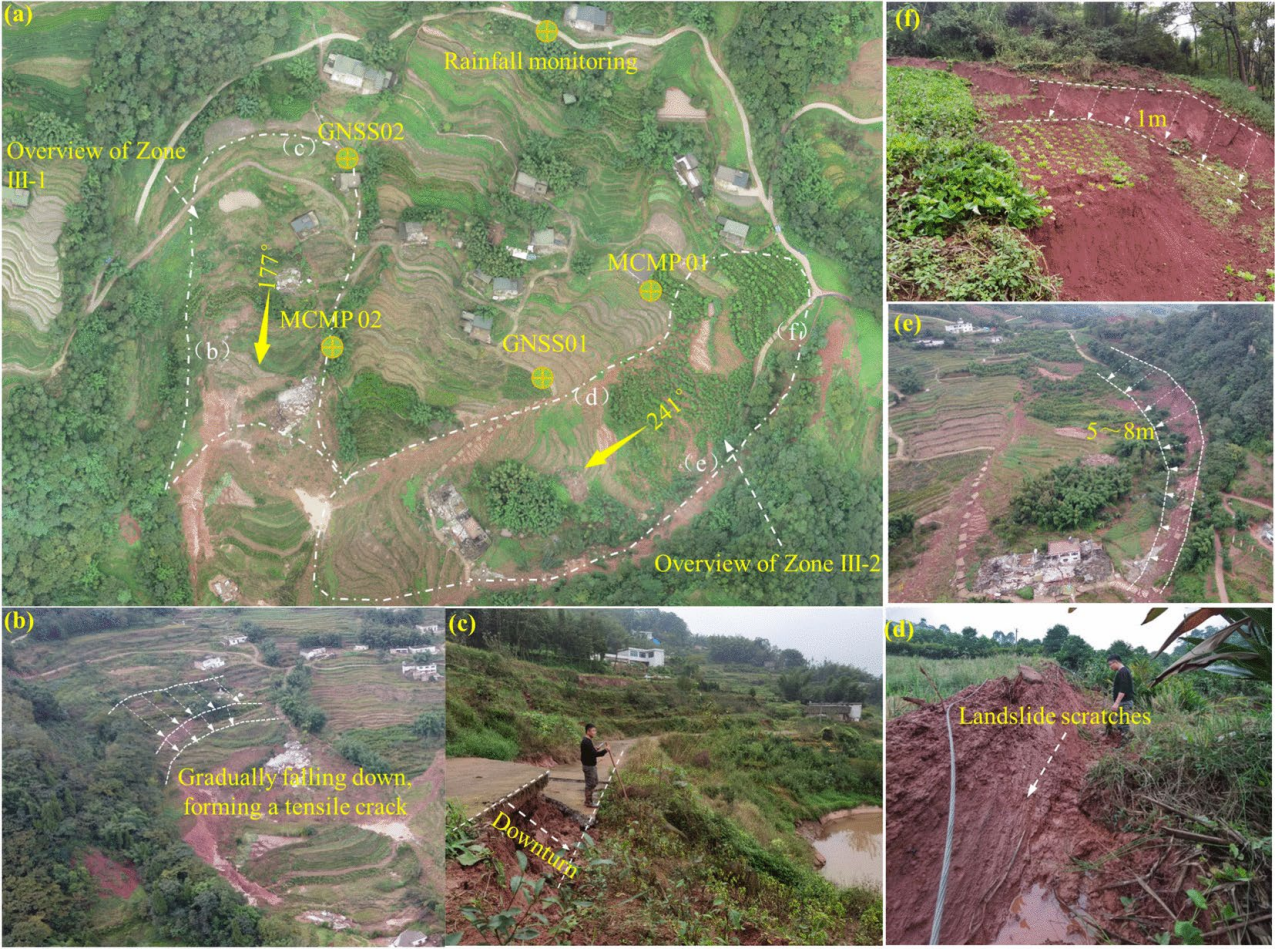
**a** Overview of Zone III and the Unslipped Area; **b** Stepped Subsidence at the Front Edge of Zone III −1; **c** Severe Subsidence at the Rear Edge of Zone III −1; **d** Scratches on the Side of Zone III −2; **e** Soil Subsidence at the Boundary of Zone III −2; **f** Damage Features at the Rear Boundary of Zone III −2.

Figure 6a shows the overall view of Zone III and the non-sliding zone. Zones III-1 and III-2 form a “surrounding” structure around the non-sliding zone. Zone III-1 has a “tongue-like” planar shape, with a main sliding direction of 177° and a terrain slope of 13–16°. The total slide length is approximately 240 m, with an average width of 105 m. The sliding body is thicker at the front and thinner at the rear, with a thickness ranging from 5–12 m (average ~ 8 m) and a volume of 2.02 × 10^5^ m^3^. The front boundary is defined by the steep back wall formed after the sliding of Zone II, the rear boundary is marked by a subsidence scarp, the left boundary is defined by a gully, and the right boundary is defined by a ridge. The rear edge of the slide is at an elevation of 600 m, while the front edge is at 550 m, resulting in a relative height difference of 50 m. Zone III-1 experienced successive subsidence due to traction from Zone II (Fig. 6b), forming hundreds of ring-shaped tensile cracks. The rear scarp shows severe subsidence (Fig. 6c).

Zone III-2 also exhibits a “tongue-like” planar shape, with a main sliding direction of 241° and a terrain slope of 10–15°. The slide is approximately 330 m long, with an average width of 130 m. The sliding body is thicker at the front and thinner at the rear, with a thickness ranging from 5–15 m (average ~ 10 m) and a volume of 4.29 × 10^5^ m^3^. The front boundary is defined by bulging soil deposits, the rear boundary by the slope crest, the left boundary by the contact line between the overburden and bedrock, and the right boundary by microtopographic ridges. The rear edge of Zone III-2 is at an elevation of 610 m, while the front edge is at 540 m, resulting in a relative height difference of 70 m (Fig. 6d). The right boundary exhibits significant scratches, while the left boundary shows noticeable soil subsidence, with depths of 5–8 m (Fig. 6e). At these locations, the bedrock, primarily purple-red mudstone, is exposed. The rear edge subsided, forming a 1-m-high steep scarp (Fig. 6f). The non-sliding zone, located between Zones III-1 and III-2, shows no visible signs of deformation based on field investigations.

According to Table 1, significant differences exist among the sliding zones in terms of sliding direction, length, width, volume, and slope gradient. Zone I has the longest length, the largest volume, and the gentlest slope gradient. Zone II has a smaller volume and the mildest slope gradient. Zone III-1 is the shortest with the smallest volume but the steepest slope gradient. Zone III-2 deviates in sliding direction from the other zones, with moderate volume and length but a more variable slope gradient. These differences may be closely related to the topographic features of the landslide, the rock layer structure, and the failure mechanisms of the landslide.

**Table 1:** Statistical Table of Basic Characteristics of Different Sliding Zones.

Sliding Zone	Main Sliding Direction(°)	Length(m)	Width(m)	Volume(m^3^)	slope angle(°)
Zone I	206	450	100	900,000	10 ~ 13°
Zone II	187	300	120	212,000	10 ~ 12°
Zone III-1	177	240	105	202,000	13 ~ 16°
Zone III-2	241	330	130	429,000	10 ~ 15°

##  Methodology

### Theoretical background

Given the differences in soil strength and free-face conditions during the failure process of traction-tensile-cracking-sliding type multi-stage landslides, this study modified the related calculation formulas of the traditional transfer coefficient method to make it more suitable for the stability analysis of multi-stage landslides. The calculation of the stability coefficient for multi-stage landslides is divided into two parts: when calculating Zone I, the thrust effect of Zone II on Zone I is considered; when calculating Zone II and higher zones, both the loss of the previous landslide section and the thrust effect of the subsequent landslide section are considered. Assuming there are m stages in the multi-stage landslide, the calculation formulas for the stability coefficient of each stage are as follows:

1 $$\left\{ \begin{gathered} F_{s}^{k} = \frac{{\sum\limits_{i = 1}^{n - 1} {(R_{i}^{k} \prod\limits_{j = i}^{n - 1} {\psi_{j}^{k} ) + R_{n}^{k} } } }}{{\sum\limits_{i = 1}^{n - 1} {(T_{i}^{k} \prod\limits_{j = i + 1}^{n} {\psi_{j}^{k} ) + T_{n}^{k} + T^{k + 1} } } }} \, k = 1 \hfill \\ F_{s}^{k} = \frac{{\sum\limits_{i = 1}^{n - 1} {(R_{i}^{k} \prod\limits_{j = i}^{n - 1} {\psi_{j}^{k} ) + R_{n}^{k} - \left| {R^{k - 1} } \right|} } }}{{\sum\limits_{i = 1}^{n - 1} {(T_{i}^{k} \prod\limits_{j = i + 1}^{n} {\psi_{j}^{k} ) + T_{n}^{k} + T^{k + 1} } } }} \, k> 1 \hfill \\ \end{gathered} \right.$$

where:

2 $$\left\{ \begin{gathered} T_{i} = W_{i} \sin \alpha_{i} \, i \ne n \hfill \\ T_{i} = W_{i} \sin \alpha_{i} + \frac{1}{2}\gamma_{{\text{w}}} Z^{2} \, i = n \hfill \\ \end{gathered} \right.$$

3 $$\psi_{i} = \cos (\alpha_{i - 1} - \alpha_{i} ) - \sin (\alpha_{i - 1} - \alpha_{i} )\tan \varphi_{i}$$

4 $$R_{i} = W_{i} \cos \alpha_{i} \tan \varphi_{i} + c_{i} l_{i}$$

where: *W*_*i*_ represents the weight of the i-th block; *ψ*_*i*_^*k*^ is the transfer coefficient of the *i*-th block in the *k*-th level landslide; *α*_*i*_ is the inclination angle of the i-th block; *γ*_w_ is the unit weight of water; *R*^k−1^ is the resistance of the *k*−1-th level landslide to the *k*-th level landslide; *T*^k+1^ is the thrust of the *k* + 1-th level landslide on the k-th level landslide; *l*_*i*_ is the length of the *i*-th block; *c*_*i*_ is the residual cohesion of the landslide soil; *φ*_*i*_ is the residual internal friction angle of the landslide soil; Z is the depth of the tensile crack. The calculation formulas for *R*^*k*−1^, *T*^*k*+1^ and Z are as follows:

5 $$T_{k - 1} = \cos (\alpha_{1}^{k} - \alpha_{n}^{k + 1} )E_{n}^{k + 1}$$

6 $$R^{k - 1} = - E_{n}^{k}$$

7 $$Z = \frac{2c}{{\upgamma \sqrt {K_{{\text{a}}} } }}$$

8 $$K_{{\text{a}}} = \tan^{2} (45^\circ - \frac{\varphi }{2})$$

where: *E*_*n*_^*k*^ represents the residual downslope force of the *i*-th block in the *k*-th level landslide. When *E*_*n*_ = 0, the inter-block downslope force equals the anti-sliding force, indicating that the landslide is in a state of limit equilibrium. When *E*_*n*_ > 0, the inter-block downslope force exceeds the anti-sliding force, and the landslide is unstable. When *E*_*n*_ < 0, the inter-block downslope force is less than the anti-sliding force, and the landslide is stable. The calculation formula for *E*_*n*_ is as follows:

9 $$\begin{gathered} E_{i}^{k} = W_{i}^{k} \sin \alpha_{i}^{k} - W_{i}^{k} \cos \alpha_{i}^{k} \tan \varphi_{i}^{kresid} - c_{i}^{kresid} l_{i}^{k} + \psi_{j}^{k} E_{i - 1}^{k} - \frac{1}{2}\gamma_{w} Z^{2} \, i = 1 \hfill \\ E_{i}^{k} = W_{i}^{k} \sin \alpha_{i}^{k} - W_{i}^{k} \cos \alpha_{i}^{k} \tan \varphi_{i}^{kpeak} - c_{i}^{kpeak} l_{i}^{k} + \psi_{j}^{k} E_{i - 1}^{k} \, 2 \le i \le n - 1 \hfill \\ E_{i}^{k} = W_{i}^{k} \sin \alpha_{i}^{k} - W_{i}^{k} \cos \alpha_{i}^{k} \tan \varphi_{i}^{kresid} - c_{i}^{kresid} l_{i}^{k} + \psi_{j}^{k} E_{i - 1}^{k} \, i = n \hfill \\ \end{gathered}$$

where: *W*_*i*_^*k*^ is the weight of the *i*-th block in the *k*-th level landslide; *α*_*i*_^*k*^ is the dip angle of the *i*-th block in the *k*-th level landslide; *E*_*i*−1_ is the residual downslope force of the *i-*1-th block; *c*_*i*_^*k*peak^、*φ*_*i*_^*k*peak^ are the peak strength parameters of the sliding block; *c*_*i*_^*k*resid^、*φ*_*i*_^*k*resid^ are the residual strength parameters of the sliding mass; *γ*_w_ is the unit weight of water.

### Numerical simulation

This study employed the particle flow discrete element method (DEM) to analyze the failure mechanism of the Wachangwan landslide, considering the softening effects of the slope under rainfall conditions, the reduction of soil strength parameters after landslide failure, and the interaction between the landslide body and the sliding bed. Based on previous numerical simulation studies of landslides^24^, the linear parallel bond model was selected as the contact model between particles^25,26^.

**Fig. 7:**
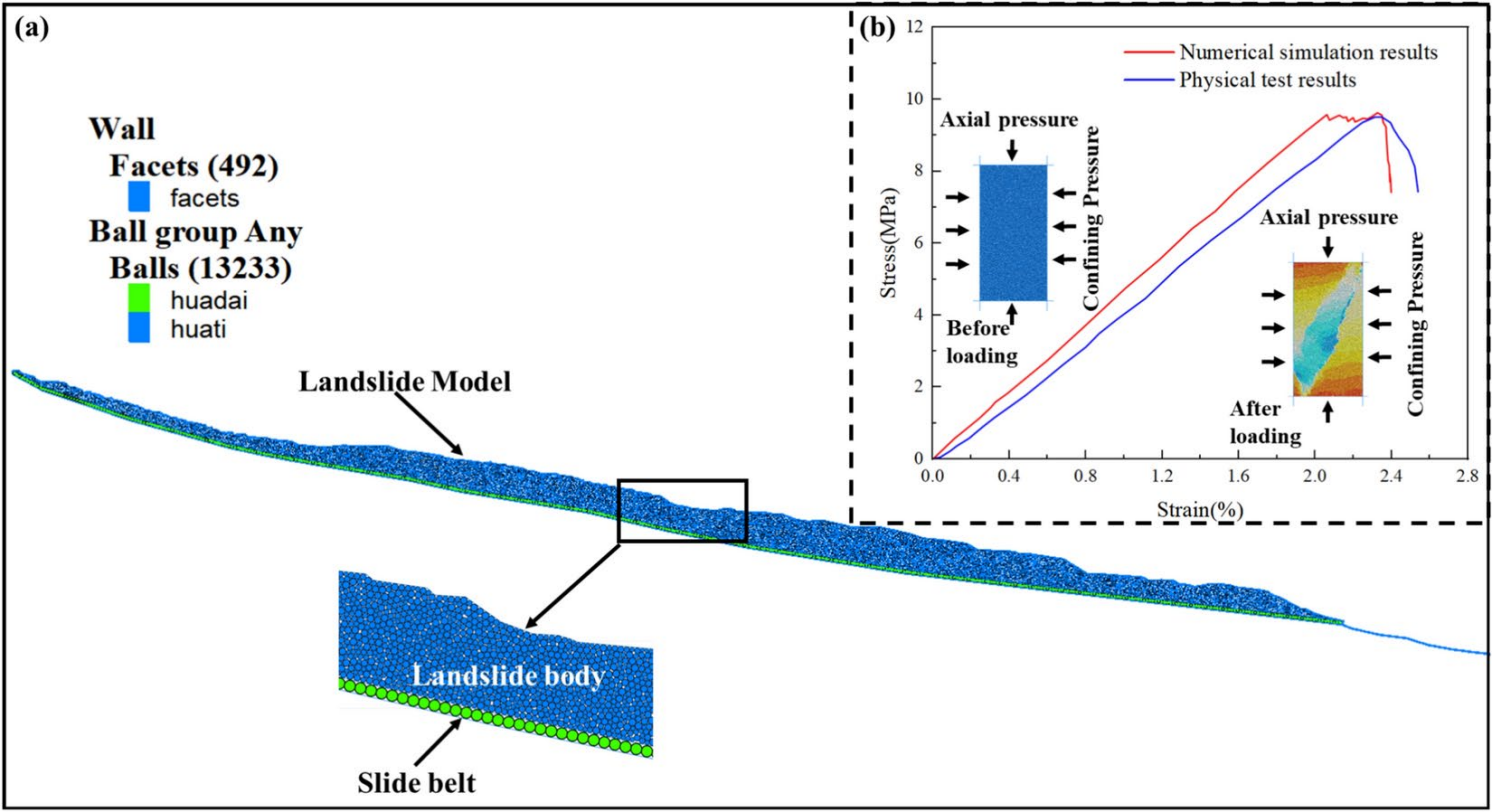
Numerical model schematic and parameter calibration results. **a** Slope model used in the DEM simulation; **b** Comparison between numerical simulation of compression tests and physical experimental results.

The simulation process involved generating spherical particles and ensuring initial stress equilibrium, followed by reducing the material parameters of the model and separately considering the landslide body and the sliding zone^27^. To analyze the failure mechanism of the landslide and its potential subsequent failure modes, the landslide model was designed based on Section Composition and structure of the landslide body. The final model is shown in Fig. 7a. The calculation was terminated when the unbalanced force reached 1e-5.

 Since the particle flow DEM software uses microscopic parameters for calculations, these parameters (e.g., particle stiffness, bond strength, friction coefficient, particle size) needed to be calibrated against the macroscopic parameters of the landslide (e.g., internal friction angle *φ*, cohesion *c*, density *ρ*).

Since rainfall is the primary triggering factor for slope movement, the permeability coefficient plays a significant role in the soil’s behavior. Based on laboratory tests, the permeability coefficient of the sliding mass was determined to be 2.5 × 10^−6^ cm/s. Additionally, the physical and mechanical parameters of the slope soil in a saturated state were calibrated to simulate continuous rainfall conditions. The physical parameters of the Wachangwan landslide are listed in Table 2. The parameters used in the DEM model were determined based on numerical simulations and biaxial compression tests, with the comparison between numerical and experimental uniaxial test results shown in Fig. 7b. The detailed parameters of the model test particles are presented in Table 3.

**Table 2:** Macroscopic Mechanical Parameters of the Landslide Mass.

Soil Sample Condition	Water content(%)	Density(g/cm^3^)	Cohesion(c/kPa)	Internal Friction Angle (φ/°)
Natural	15.61	1.95	22.58	25.62
Saturated	34.57	2.27	20.06	21.34

**Table 3:** Microscopic Parameters of the Landslide Body Model.

	Particle Density/(kg·m^−3^)	Particle Size/m	Normal Stiffness/(N·m^−1^)	Tangential Stiffness(N·m^−1^)	Normal Bond Strength/N	Tangential Bond Strength/N	Friction Coefficient	Damping
Landslide Body	2250	0.2 ~ 0.5	5e7	5.0e7	1.5E4	1.5E4	0.36	0.4
Slip Zone	2250	0.8	5e7	5.0e7	1.5E4	1.5E4	0.31	0.4

## Results and discussion

### Failure mode and deformation mechanism analysis

**Fig. 8:**
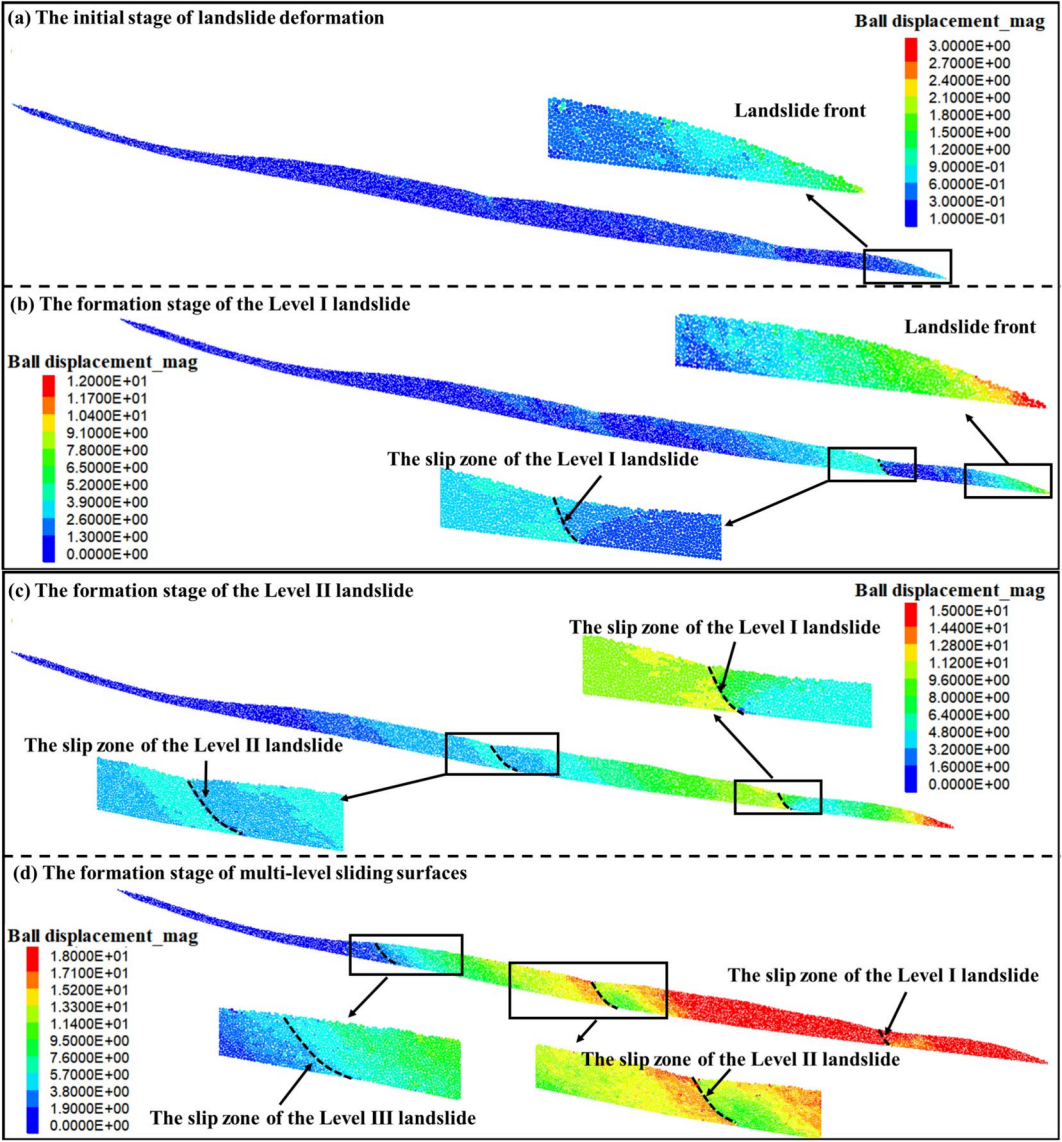
Numerical simulation process of the multi-stage evolution of the Wachangwan landslide.

Figure 8 illustrates the displacement cloud map of the Wachangwan landslide at different time intervals. At the calculation step of 2.5 × 10^4^, significant deformation occurred in the front edge of the landslide, with a maximum localized deformation reaching 1.5 m (as shown in the displacement cloud map in Fig. 8a a of the landslide front). Other areas of the landslide showed relatively smaller deformations. The primary reason for this phenomenon lies in the composition of the landslide material, which is mainly silty clay with relatively low permeability. After rainfall, since the soil at the front edge is located at the lower part of the slope, rainwater tends to accumulate in this area, causing the soil to reach saturation. In a saturated state, the water content of silty clay increases significantly, leading to a rise in pore water pressure, which directly reduces the effective stress of the soil. Consequently, localized deformation occurs on the slope surface, indicating the landslide is in its early deformation stage (as shown in Fig. 8a).

As rainfall continues, tensile cracks are prone to develop on the surface of the landslide body. The expansion of these cracks further accelerates the infiltration process of rainwater. During this process, the strength-softening effect and hydrostatic pressure induced by rainwater become increasingly significant, leading to a gradual deterioration of the soil’s physical and mechanical properties. When the rainfall exceeds the natural infiltration capacity of the soil, excess water forms surface runoff on the slope, which not only disrupts the original slope morphology but also intensifies toe erosion, eventually triggering localized instability.

At the calculation step of 7.5 × 10^4^, as rainwater continues to infiltrate deeper into the slope, the infiltration front gradually advances inward. The soil regions it passes through rapidly reach saturation, causing a significant reduction in the physical and mechanical properties of the soil within the slope’s front margin. Meanwhile, the increase in soil density after saturation leads to a greater self-weight of the slope. The natural soil moisture content is 15.16%, whereas the saturated soil moisture content reaches 34.57%. This rise in moisture content significantly accelerates the forward shift of the slope’s center of gravity. When the infiltration front finally reaches the sliding surface, the soil above the sliding surface becomes fully saturated. At this stage, the permeability coefficient of the soil in the slope’s front decreases, hindering water drainage, further deteriorating the physical and mechanical properties of the sliding zone soil. Under the combined effects of increased self-weight, weakened soil strength, and seepage forces, the primary slip surface eventually forms (Fig. 8b). The sliding failure in Zone I disrupted the original equilibrium of the colluvial slope. After the formation of the primary sliding surface, a steep scarp formed at the rear of Zone I, creating a favorable free face condition for the sliding in Zone II. At the calculation step of 15 × 10^4^, influenced by multiple factors such as groundwater flow and the self-weight of the soil mass, Zone II experienced sliding along the free face formed at the rear of Zone I, which resulted in the formation of the secondary sliding surface. This movement also triggered a progressive subsidence of the soil masses on both sides of Zone II’s rear boundary (Fig. 8c).

As the calculation steps further advanced to 23 × 10^4^, the loss of anti-sliding resistance in the rear of Zone II ultimately led to the sliding of Zone III along the steep scarp at the rear of Zone II (Fig. 8d). Based on the above analysis, it is evident that the final simulation results demonstrating the landslide failure characteristics align closely with the failure features observed in the field investigation of the Wachangwan landslide (as illustrated in Figs. 4,5,6).

 Field investigations combined with numerical simulations reveal that the failure mechanism of the Wachangwan landslide exhibits a multi-stage progressive instability pattern influenced by episodic heavy rainfall. This process is primarily characterized by unloading at the slope’s front edge, which triggers successive rearward traction-induced sliding. Specifically, the landslide’s front edge initially experienced movement due to the saturation of the soil under prolonged heavy rainfall, leading to the formation of the first-level sliding zone (Zone I). Subsequently, the rear area began deforming as the front zone lost its anti-sliding support, which resulted in the formation of the second-level sliding zone (Zone II). When the front of the third-level sliding zone (Zone III) lost the resistance from Zone II, both Zone III-1 and Zone III-2 slid under their self-weight along the free face formed by the steep scarp at the rear of Zone II. After the failure of Zone III-1 and Zone III-2, loose soil primarily accumulated at the front of the non-sliding zone. This accumulation unexpectedly increased the anti-sliding resistance of the non-sliding zone, preventing overall slope failure in the final stage.

**Fig. 9:**
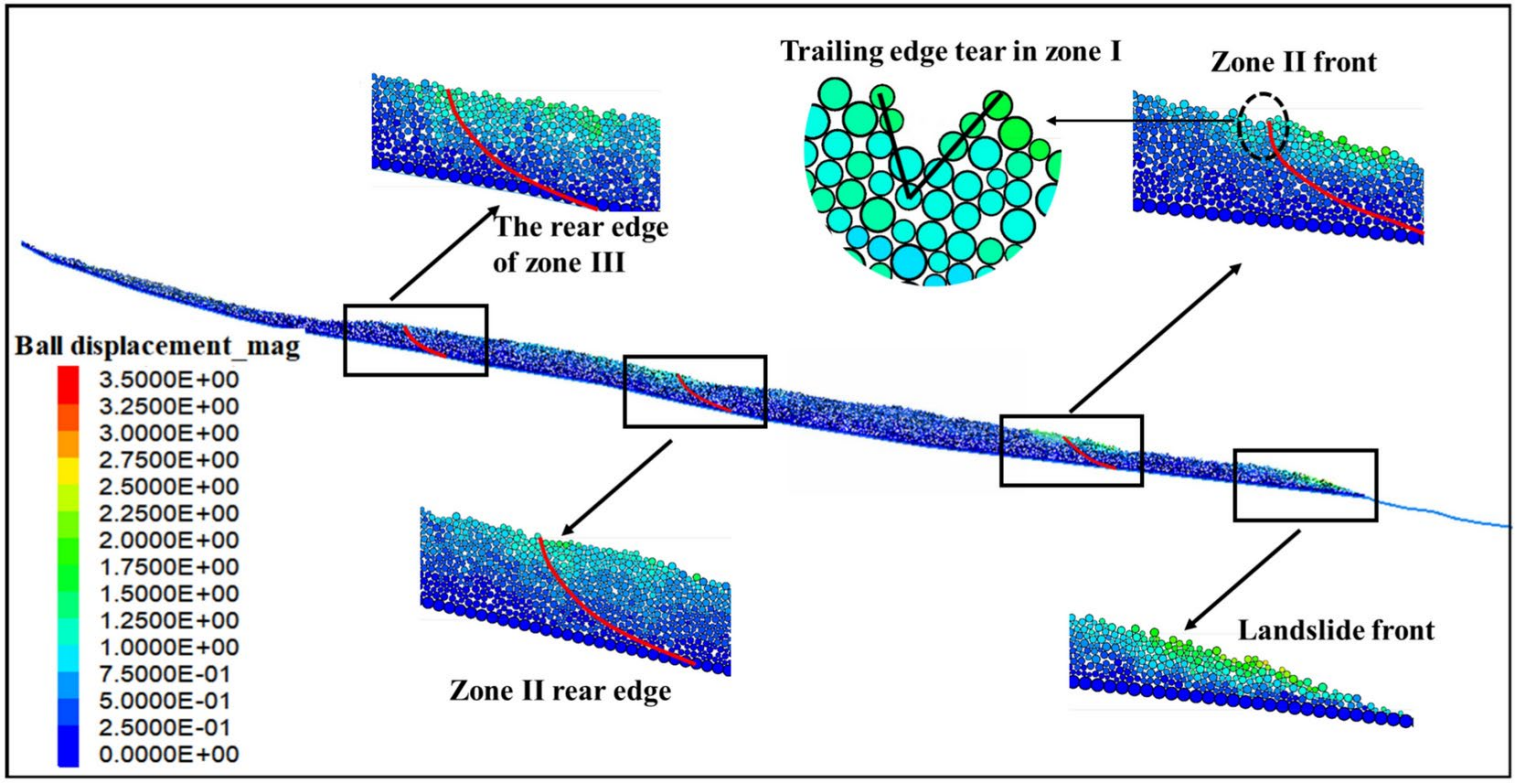
Deformation displacement cloud map of the Wachangwan landslide under subsequent rainfall conditions.

To further investigate the specific impact of subsequent heavy rainfall on the Wachangwan landslide, a simulation analysis was conducted focusing on the slope’s post-failure condition. Figure 9 presents the displacement cloud map of the Wachangwan landslide under continued rainfall. The analysis reveals that the slope remains susceptible to further instability under sustained rainfall, with the failure mode consistently exhibiting the characteristic traction-tension-sliding pattern, along with distinct segmented and staged sliding features.

Initially, the soil mass at the slope toe was the first to experience movement, with a significant displacement ranging between 1.5 to 2.0 m. Subsequently, the rear edge of Zone I in the lower slope also showed significant displacement, accompanied by tension cracks, with a displacement range of 1.0 to 1.8 m. Similarly, the rear edge of Zone II displayed visible displacement, gradually penetrating the slip zone, with a particle displacement range of approximately 1.0 to 1.6 m.

Additionally, the ongoing rainfall induced minor movement on the slope surface, with displacement values ranging from 0.15 to 0.50 m. Particles at the rear of the sliding zones exhibited tension cracking and movement under the traction of the sliding mass. As rainfall continued to intensify, the plastic zone near the rear of Zone I further penetrated the slip zone, triggering sliding along the slip surface. With the sliding of Zone I, Zone II developed favorable free face conditions, and its rear arc-shaped plastic zone also penetrated the slip zone and initiated sliding. Finally, the front edge of Zone III developed a free face, while the rear edge also experienced sliding failure. Notably, under continued rainfall, the primary areas of sliding failure in the Wachangwan landslide remained concentrated at the slope toe and the rear edges of Zones I and II, whereas the non-sliding zone maintained relative stability.

### ***Stability analysis***

The formation of traction-tension-crack-sliding multi-level landslides starts from the foremost front, where the slope toe serves as the first-level landslide, extending backward to the *k*-th level landslide. Using the aforementioned stability calculation method, the stability of the Wachangwan landslide was evaluated. The saturated unit weight of the sliding zone soil is 22.5 kN/m^3^, and the soil strength parameters were obtained through laboratory shear tests, as shown in Table 4.

**Table 4:** Strength Parameters of the Sliding Zone Soil in the Wachangwan Landslide.

Type	Cohesion(c/kPa)	Internal Friction Angle (φ/°)
Peak Strength	12.75	11.87
Residual Strength	10.63	10.06

**Fig. 10:**
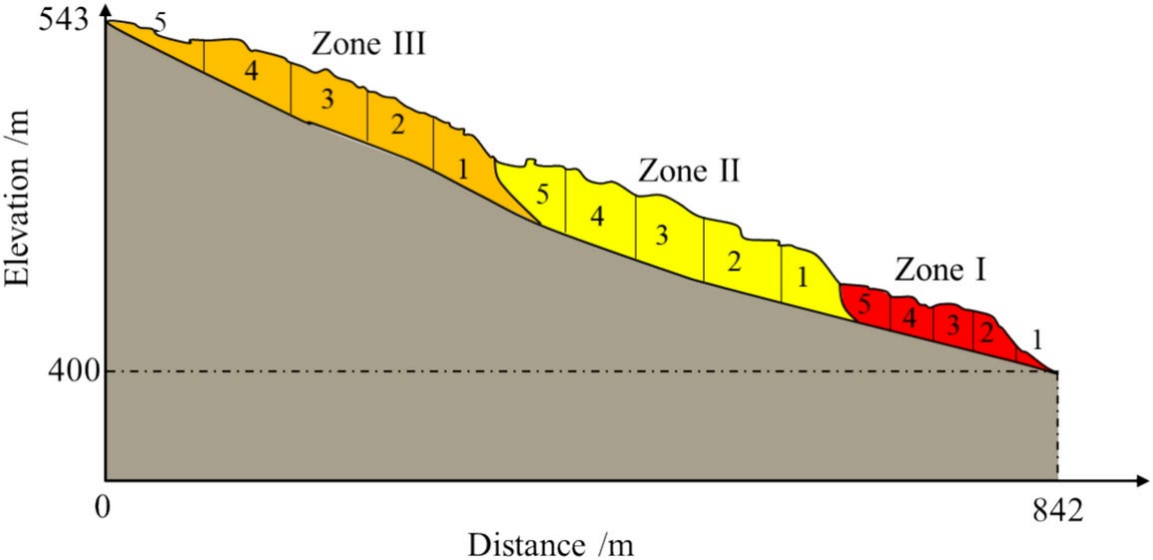
Block Division and Calculation Diagram of the Wachangwan Landslide.

In general, accurately determining the failure surface has a significant impact on the precision and reliability of landslide stability analysis. In this study, the failure surface of the Wachangwan landslide was determined by conducting drilling observations at different locations on the landslide surface, combined with the distribution of soil layers and the characteristics of weak planes. Additionally, to simplify the calculations, it was assumed that the landslide experienced multiple heavy rainfalls prior to failure, which caused the infiltration line to reach the slip surface, resulting in the soil above the slip surface being in a saturated state. During the calculation process, the post-failure Wachangwan landslide was evaluated based on the calculation profile in Fig. 3b and numerical analysis results. Each level of the landslide was divided into five blocks, with the division scheme shown in Fig. 10. According to the above calculation method, the first and last blocks of each landslide level used the residual strength of the landslide soil, while the remaining blocks used the peak strength. The stability factors calculated using formulas (1) to (4) and the specific residual sliding force calculations for each level of the landslide are detailed in Table 5.

**Table 5:** Stability Calculation Process Considering the Impact of Multi-Level Landslides.

Landslide Level	Block Number	α/(°)	*W*_*i*_/(kN/m^−1^)	*ψ*_*i*_^*k*^	*T*_*i*_/(kN/m^−1^)	*R*_*i*_/(kN/m^−1^)	*ψ*_*i*_*E*_i-1_/(kN/m^−1^)	Stability Coefficient
Zone I	1	21.00	578.13	/	207.18	207.47	/	0.992
	2	19.00	2120.74	0.997	896.22	793.04	147.99	
	3	18.00	6009.63	0.960	2750.39	2236.85	651.06	
	4	18.00	5750.62	0.954	4417.24	3350.10	1287.99	
	5	15.00	16,128.69	0.989	8388.46	6397.24	2410.64	
Zone II	1	16.00	18,502.65	/	13,399.10	9921.81	/	0.994
	2	10.00	47,953.43	0.985	21,264.90	18,978.72	3349.43	
	3	13.00	57,428.23	1.016	33,860.38	29,929.62	5623.78	
	4	12.00	74,544.05	1.015	49,907.94	44,643.08	7760.25	
	5	10.00	66,914.94	1.016	62,289.45	58,284.20	7119.73	
Zone III	1	10.00	64,012.43	/	74,415.00	72,324.61	/	1.004
	2	9.00	5570.60	1.014	76,492.93	74,948.93	5368.65	
	3	8.00	2795.21	1.010	77,953.48	76,916.00	4935.16	
	4	12.00	1639.88	0.985	79,069.24	78,273.76	4748.94	
	5	8.00	3389.31	1.002	78,369.83	78,260.30	4028.02	

The calculation results indicate that the stability coefficients of each landslide level increase progressively, confirming that the landslide occurs in a stepwise, segmented manner from the slope foot to the slope top, characteristic of a typical multi-level landslide. Under heavy rainfall conditions, the stability coefficients of Zones I and II are less than 1, indicating an unstable state, while the stability coefficient of Zone III is 1.004, suggesting a marginally stable state. Therefore, under subsequent heavy rainfall conditions, the I and II zones of the Wachangwan landslide are likely to continue sliding. However, the III zone has a lower likelihood of further sliding due to the absence of thrust from the preceding slide and its gentler slope. This observation is consistent with the calculated safety factor results.

The final stability coefficient calculation method, after being improved, provides a solid theoretical foundation for the subsequent management and implementation of the Wachangwan landslide control plan. Moreover, considering that rainwater tends to accumulate at tensile cracks on the slope surface and generate hydrostatic pressure, this factor is particularly important in the force analysis of multi-stage landslides under rainfall conditions. Therefore, the optimized transfer coefficient method can accurately assess the stability coefficient of each stage of the landslide under rainfall conditions, offering an effective basis for evaluating the stability of rain-induced landslides. It also provides more precise design parameters for formulating targeted landslide mitigation measures and explores a new approach for evaluating the stability of multi-stage landslides.

However, it is worth noting that due to the multi-stage failure characteristics of the landslide and the differences in the mechanical parameters of each sliding zone, the calculated stability coefficients may tend to be conservative, i.e., the values may be somewhat lower. In addition, the landslide’s actual movement is influenced by multiple complex factors, such as dynamic changes in groundwater, rainfall intensity, and the anisotropy of the sliding surface soil. Therefore, while this method provides valuable predictions, there may be some discrepancy between the predicted results and the actual development of the landslide. Given this, future improvements and optimizations of this algorithm should be carried out through continuous practical verification and adjustments based on real-world situations.

### Displacement analysis of monitoring points

**Fig. 11:**
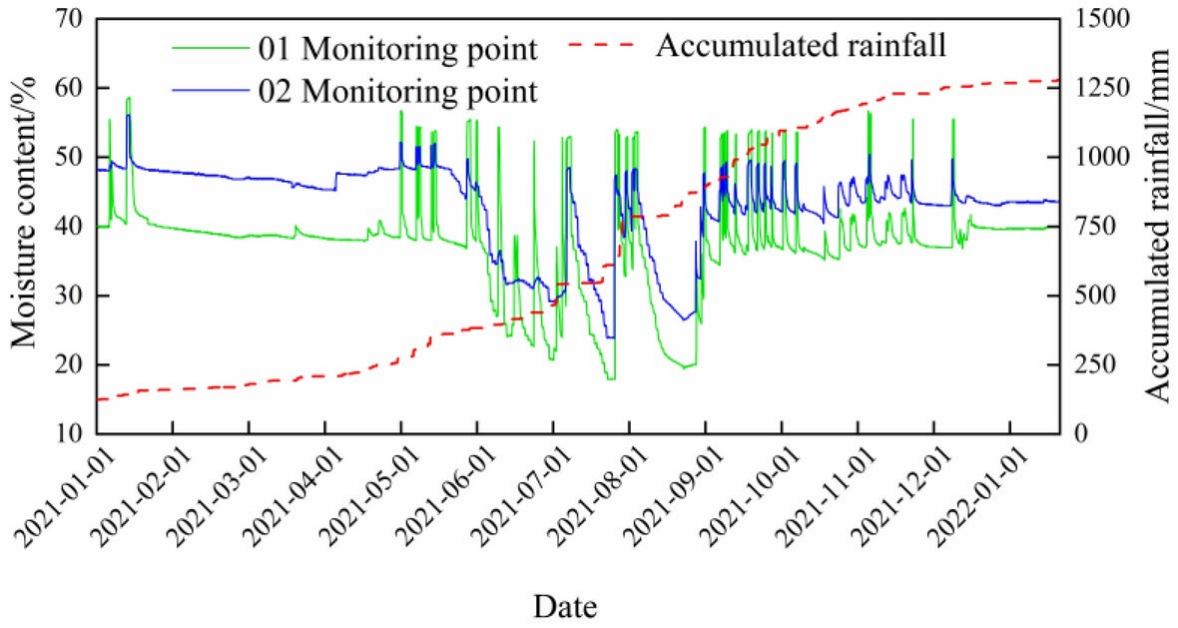
Moisture Content and Cumulative Rainfall Curves at Monitoring Points (2021–2022).

**Fig. 12:**
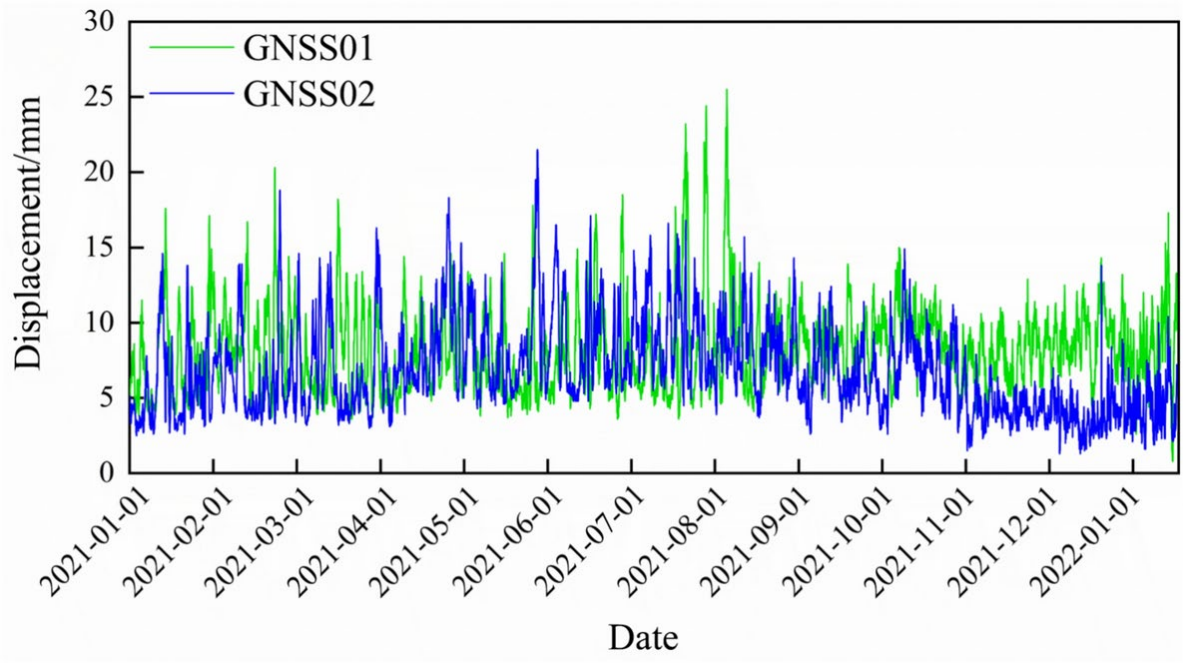
Cumulative Displacement Curves at Monitoring Points (2021–2022).

To study the influence of Zones III-1 and III-2 on the un-slid zone between them, monitoring points were established along the boundary of the un-slid zone to measure soil moisture content, rainfall, and displacement, aiming to observe deformation in the un-slid zone. Figure 11 presents the cumulative variation curves of soil moisture content and rainfall recorded at the monitoring points from January 2021 to January 2022, while Fig. 12 shows the displacement variation curves at the boundary of the un-slid zone near Zone III.

 From Figs. 11 and 12, it can be observed that the rainfall in 2021 was concentrated between May and September. Due to the summer temperatures and rainy season, the soil moisture content experienced significant changes during this period. However, the displacement monitoring points did not show any notable trend of deformation throughout the monitoring period. The fluctuations in the data were mainly caused by GNSS monitoring accuracy and external interference.

 This indicates that, although rainfall increased the soil moisture content, the un-slid zone remained relatively stable. This stability is attributed to the unique location of the un-slid zone—situated between Zones III-1 and III-2. The loose soil mass primarily accumulates at the front edge of the unmoved zone, causing lateral pressure on both sides of the rear boundary of the unmoved zone due to the sliding mass from the sliding zone. This lateral pressure helps maintain the basic stability of the unmoved zone, which is consistent with the results simulated in Fig. 9.

Thus, the stress state of the slope at its specific location is also a crucial factor influencing slope movement.

## Conclusions

This study focuses on the Wachangwan landslide and, based on detailed field investigations, systematically examines its engineering geology and deformation characteristics through numerical simulations. The main conclusions are as follows:

1.The Wachangwan landslide can be divided into four zones—Zone I, Zone II, Zone III-1, and Zone III-2—based on differences in geological features, shear-out locations, deformation characteristics, and deformation sequences.

2.The deformation and failure of the Wachangwan landslide are primarily caused by prolonged heavy rainfall, which saturates the landslide soil at the front edge. The infiltration of large amounts of surface water into the soil body causes sliding failures along the steep free face at the front edge. The destabilized rear soil, losing support due to the free face, undergoes subsequent sliding, leading to stepwise pulling and deformation, ultimately forming a multi-stage landslide.

3.Using discrete element simulation software to analyze the deformation mechanism and subsequent evolution of the Wachangwan landslide under rainfall conditions, it was observed that future sliding failures would mainly occur at the landslide tongue, the rear edge of Zone I, and the rear edge of Zone II. The failure mode of the landslide is characterized by a traction-tensile-shear mechanism.

4.Based on the traditional transfer coefficient method, an improved algorithm for calculating the stability of multi-stage landslides under rainfall conditions was developed. Stability calculations for the Wachangwan landslide at its current stage revealed stability coefficients of 0.992, 0.994, and 1.004 for Zones I, II, and III, respectively. These results provide a new approach to evaluating the stability of multi-stage landslides.

## Data Availability

The data that support the findings of this study are available from the corresponding author upon reasonable request.
